# Relationship between admission serum uric acid to lymphocyte ratio and the risk of ischemic stroke recurrence and death: a prospective study

**DOI:** 10.7189/jogh.15.04240

**Published:** 2025-09-12

**Authors:** Chenning Song, Guangxiao Li, Yuanmeng Tian, Li Jing, Dong Chen, Weizhong Wang, Yonggang Shi, Dongyu Wang, Dongqun Li, Xinbin Hao, Liying Xing, Shuang Liu

**Affiliations:** 1 Institute of Preventive Medicine, China Medical University, Shenyang, People’s Republic of China; 2 Department of Chronic Disease, Liaoning Provincial Center for Disease Control and Prevention, Shenyang, People’s Republic of China; 3 Department of Medical Record Management Center, the First Hospital of China Medical University, Shenyang, Liaoning, People’s Republic of China; 4 Department of Neurology, Central Hospital of Da Lian City, Da Lian, Liaoning, People’s Republic of China; 5 Department of Neurology, Central Hospital of Dan Dong City, Dan Dong, Liaoning, People’s Republic of China; 6 Department of Neurology, Central Hospital of Dong Gang City, Dan Dong, Liaoning, People’s Republic of China; 7 Department of Neurology, Central Hospital of Jin Zhou City, Jin Zhou, Liaoning, People’s Republic of China; 8 Department of Neurology, Central Hospital of Ying Kou City, Ying Kou, Liaoning, People’s Republic of China; 9 Department of Neurology, Central Hospital of Liao Yang City, Liao Yang, Liaoning, People’s Republic of China; 10 Department of Ultrasound, the Fourth Affiliated Hospital of China Medical University, Shenyang, Liaoning, People’s Republic of China

## Abstract

**Background:**

This study aims to explore the relationship between the Serum uric acid to lymphocyte ratio (ULR) level at admission and the long-term risk of recurrence and death in patients with acute ischemic stroke (AIS) in Liaoning Province.

**Methods:**

This multicentre prospective study enrolled 7966 subjects who experienced ischemic stroke (IS) across Liaoning Province, China. The Cox proportional hazards model and Kaplan-Meier curves were used to explore the association of ULR with the risk of recurrence and death in IS.

**Results:**

During a median follow-up period of 4.08 (3.35, 4.43) years, there were 1311 cases of stroke recurrence or death, 1429 cases of cardiovascular (CVD) death, and 910 cases of stroke-cause death. In analysis comparing the Q4 and Q1group, after multivariate adjustment, ULR was significantly positively associated with the incidence of stroke recurrence or death (Q4 *vs*. Q1: HR = 1.21; 95% CI = 1.04, 1.42), CVD death (Q4 *vs*. Q1: HR = 1.16; 95% CI = 1.00, 1.34), and stroke-cause death (Q4 *vs*. Q1: HR = 1.26; 95% CI = 1.05, 1.52). Additionally, the significant correlation between ULR and the risk of IS recurrence or death was partially mediated by diastolic blood pressure (DBP) (8.53%) and systolic blood pressure (SBP) (3.59%) in a positive manner.

**Conclusions:**

This study demonstrates that higher ULR is significantly associated with an increased risk of recurrence and death following IS. The findings suggest that ULR could serve as a valuable prognostic marker in clinical practice, particularly in managing patients with IS.

Stroke is an acute and focal neurological deficit caused by cerebrovascular (CVD) issues [1] representing the third leading global cause of disability and mortality [2], imposing a heavy burden, particularly in low- and middle-income countries (LMICs) [2–4]. Ischemic stroke (IS), the most common type, has high prevalence, recurrence, disability, and mortality rates [3-6], highlighting the need for active prevention and research.

Systemic inflammation plays a critical role in stroke onset and progression [7–9], contributing to vascular injury, thrombogenesis, and neural damage [7–10]. Two inflammation-related biomarkers – serum uric acid (SUA), linked to oxidative stress [10–12], and lymphocyte count, reflecting immune regulation [13–15] – are widely used.

The serum uric acid-to-lymphocyte ratio (ULR) is a novel composite inflammatory marker reflecting the balance between oxidative stress and immune suppression. It has shown potential prognostic value in malignancies [16] and CVD disease [17]. Emerging evidence suggests that ULR may be more strongly associated with stroke risk than either SUA or lymphocyte count alone [18].

However, prospective studies assessing the association between ULR and adverse outcomes, such as recurrence and death, in patients with IS are limited. Elevated ULR may indicate underlying mechanisms including oxidative stress, endothelial dysfunction, and impaired immune surveillance during the acute and subacute phases of IS.

This study aims to evaluate whether higher ULR levels at admission are independently associated with increased risk of recurrence and mortality in IS patients, using a prospective cohort design to support risk stratification and personalised management strategies.

## METHODS

### Data analysis

From January 2019 to December 2020, a total of 10 405 patients with acute ischemic stroke (AIS) were continuously enrolled in eight tertiary hospitals across seven cities (Shenyang, Dalian, Dandong, Jinzhou, Yingkou, Liaoyang, and Chaoyang) in Liaoning Province. The diagnosis of AIS was made in accordance with the World Health Organization (WHO) criteria and further confirmed by magnetic resonance imaging (MRI) or computed tomography (CT). The study was registered at the China Clinical Trial Registration Center (ChiCTR1900021284).

### Participants

Inclusion criteria were:

1) Initial or recurrent AIS confirmed by CT/MRI, hospitalised within 72 hours of onset;

2) There were one main nervous system symptoms and signs (hemiplegia involving more than two body parts, numbness involving one side of more than two body parts, homonymous hemianopia, aphasia) or two secondary nervous system symptoms and signs (diplopia, vertigo or gait abnormality, dysarthria or dysphagia or dysphonia) and lasted for more than 24 hours;

3) Residents with permanent residence registration in the region (one year residence in the region for more than six months);

4)There is no plan to emigrate or work outside within two years;

5) Voluntary participation and signing of informed consent.

Exclusion criteria included:

1) Non-acute ischemic stroke, not diagnosed by computed tomography or magnetic resonance imaging, and hospitalised more than 72 hours after onset;

2) Non-resident residents in the region (one year residence in the region for more than six months);

3) There are plans to emigrate or work outside within two years.

**Figure 1:**
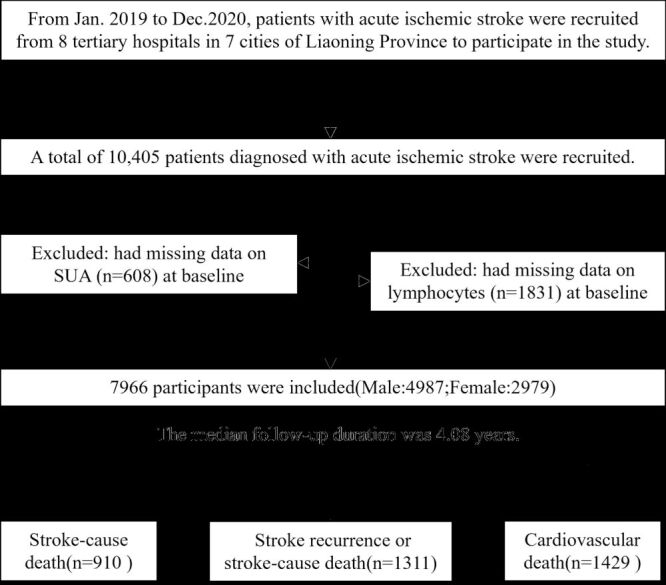
Flowchart for participants selection.

Out of the initial 10 405 AIS patients, those with missing baseline SUA data (n = 608) and those missing baseline lymphocyte data (n = 1831) were excluded. Finally, 7966 patients (76.6%) were included, with 4987 males and 2979 females (**Figure 1**). All participants provided informed consent.

### Data collection

The baseline data of the participants had been collected by well-trained neurologists using standardised clinical research forms (CRFs) through face-to-face interviews with the patients. At the time of hospital admission, general information was collected, including demographics (gender, age, place of residence, educational level, *etc*.), lifestyle, physical examination, Modified Rankin Scale (mRS) scores, and National Institutes of Health Stroke Scale (NIHSS) scores. Additional information was extracted from medical records, including clinical characteristics, medication history, and treatment status (including intravenous thrombolysis (IVT) and endovascular therapy (EVT)).

Upon admission, blood samples were collected from all participants at least eight hours after fasting. The blood samples were sent to the biochemical laboratories at each study site for laboratory measurements, including SUA, lymphocyte count, fasting blood glucose (FBG), triglycerides (TG), total cholesterol (TC), low-density lipoprotein cholesterol (LDL-C), high-density lipoprotein cholesterol (HDL-C), thrombin time (TT), prothrombin time (PT), and serum creatinine (Cr). In cases where multiple measurements were available, the initial measurement at the time of admission was considered the baseline level. Upon discharge, the mRS scores and NIHSS were reassessed, and the survival status was recorded.

The enrolled patients were followed up by trained neurologists via telephone or outpatient visits at six, 12, 24, and 36 months after discharge, to collect relevant data. In cases where communication with the patient was difficult, information was gathered from caregivers and patient medical records were reviewed to supplement the follow-up. Additionally, death data were obtained from the national population registry of the National Bureau of Statistics of China. Furthermore, a final follow-up was conducted on 26 January 2024, which included recurrence status, survival status, time of death, and underlying cause of death.

The Liaoning Provincial Center for Disease Control and Prevention served as the data coordination, quality control, and analysis centre for the study.

### Clinical outcomes and definition

The primary outcome of this study was stroke recurrence or stroke-cause death, whichever occurred first. Secondary outcomes included all-cause death and CVD death. Stroke recurrence was defined as the appearance of new neurological dysfunction symptoms or the exacerbation of existing symptoms, lasting more than 24 hours, and did not include progressive strokes. The diagnosis of stroke recurrence was made by identifying new ischemic or haemorrhagic lesions on brain CT and/or MRI scans. Stroke recurrence was defined as a new event of IS, haemorrhagic stroke (HS), or subarachnoid haemorrhage, but not a transient ischemic attack (TIA). A minimum interval of one month was required between the recurrence and the initial IS. The determination of the cause of death was based on the International Classification of Diseases, 10th Edition (ICD-10) codes listed on the death certificate, with stroke-cause deaths coded as I60-I64 and I69 in ICD-10, while deaths due to CVD were those with the cause of death coded as I20-I25, I60-I64, and I69. The final adjudication of all events was the responsibility of the Outcome Assessment Committee, which was completed through a detailed review of electronic medical records. During a median follow-up duration of 4.08 (interquartile range (IQR) = 3.35, 4.43) years, a total of 1311 (16.5%) cases of stroke recurrence or stroke-cause deaths, 2370 (29.8%) cases of stroke recurrence or all-cause deaths, 1429 (17.9%) cases of CVD deaths, and 910 (11.4%) cases of stroke-cause deaths were observed.

ULR was calculated as SUA (mg/dl)/lymphocyte count ( × 109/L) [18], as previously reported. Regular exercise was defined as participating in moderate-intensity activities at least three times a week, such as brisk walking (approximately 4–6 km/h) for at least 30 minutes each time. Patients who did not meet these criteria were categorised as lacking exercise [19]. Body mass index (BMI) was calculated as weight (kg) divided by the square of height (m). Past medical histories of stroke, coronary heart disease (CHD), hypertension, and diabetes were determined based on the patient’s self-report or their previous medical records. Atrial fibrillation (AF) was identified through electrocardiogram reports and/or a previous diagnosis of AF. Family history of stroke was obtained through patient self-report and could also be supplemented by caregivers or through reviewing medical records. Dyslipidaemia refers to TC≥6.22 mmol/L or TG≥2.27 mmol/L or LDL-C≥4.14 mmol/L or HDL-C<1.04 mmol/L or the use of lipid-regulating drugs within the last three months [20]. Blood pressure was measured by taking the average of three repeated readings.

### Statistical analysis

Baseline characteristics were presented as mean ± standard deviation for continuous variables after assessment of normality, or as median with IQR for significantly skewed variables; categorical variables were expressed as frequency with percentages. Group comparisons were performed using ANOVA or Kruskal-Wallis H test for continuous variables (according to distribution normality), and χ^2^ test for categorical variables, as appropriate. Participants were divided into four groups based on the quartiles of ULR. The Kaplan-Meier method was used to estimate the risks of different adverse outcomes in patients with IS. The incidence rate of various clinical outcomes was calculated by dividing the number of events by the total follow-up time, expressed in person-years. The Cox proportional hazards model was used to estimate the relationship between ULR quartiles and the risk of recurrence and death from IS. Prior to model fitting, the proportional hazards assumption was formally validated using Schoenfeld residual tests. Covariate selection was based on established methodologies from published literature [8,18], with the clinically significant addition of reperfusion therapy variables (IVT and EVT) to enhance the model's relevance for stroke outcomes. Three models were established: Model 1 was unadjusted; Model 2 adjusted for age, sex, education, current drinking, current smoking, lack of exercise, BMI, systolic blood pressure (SBP), FBG, dyslipidaemia, and Cr. Model 3 further adjusted for prior history of stroke, IVT, EVT, and admission mRS. Restricted cubic splines were used to evaluate the dose-response association between ULR and different outcomes.

After establishing the relationship between ULR and stroke recurrence or stroke-cause death, we employed a stepwise regression approach for mediation analysis to test whether the association between ULR (X) and stroke recurrence or stroke-cause death (Y) is mediated by other metabolic factors (M). In the mediation analysis, we adjusted for age, sex, education, current drinking, current smoking.

Power analysis was conducted using PASS, version 15.0.5 (NCSS, LLC, Kaysville, Utah, USA). Our power calculation was based on detecting a clinically meaningful difference in stroke recurrence or stroke-related mortality between the highest-risk (Q4) and lowest-risk (Q1) ULR quartiles. Assuming a two-sided log-rank test with α = 0.05 significance level and equal group allocation (n = 1992 per quartile), the study achieved 86.7% power to identify a hazard ratio (HR) of 1.30. This calculation incorporated the following prespecified parameters:

1) an anticipated 20% event rate in the Q1 reference group during the 4-year follow-up period, and

2) proportional hazards between groups. The selected HR threshold of 1.30 was determined a priori to reflect the minimum clinically important difference supported by existing literature [18].

The other statistical analysis for our study was conducted using SPSS 26.0 (IBM Corp., Armonk, NY, USA) and *R*, version 4.1.2 (R Foundation for Statistical Computing, Vienna, Austria) statistical software. All reported *P*-values were based on two-sided significance tests, and values less than 0.05 were considered statistically significant.

## RESULTS

### Baseline characteristics of the study population

In this study, a total of 7966 participants were included at baseline, of which 2979 (37.4%) were female, with an average age of 66.66 ± 11.40 years. The average admission mRS score was 1.72 ± 1.53, and the average admission NIHSS score was 3.69 ± 4.83. The baseline characteristics of the participants, classified according to quartiles of ULR (**Table 1**). As the groups progressed from Q1 to Q4, there was a significant linear upward trend in current drinking, history of medical conditions (including history of stroke, CHD, AF, hypertension, and diabetes mellitus), family history of stroke, use of lipid-lowering agents, treatment received (IVT and EVT), and laboratory indicators such as blood pressure (SBP, diastolic blood pressure (DBP)), FBG, Cr, and SUA. On the other hand, female gender, hypoglycaemic agents, TG, TC, LDL-C, TT, PT, and lymphocyte count showed a linear downward trend.

**Table 1:** Baseline characteristics and laboratory parametres of the study groups

Characteristics	Overall	Quartiles of ULR				*P* for trend
		**Q1 (≤127.8)**	**Q2 (127.9 to 179.0)**	**Q3 (179.1 to 252.4)**	**Q4 (≥252.5)**	
Participants, n (%)	7966	1992	1991	1993	1990	
Age, years (mean ± SD)	66.66 ± 11.40	66.04 ± 11.25	65.68 ± 11.52	66.77 ± 11.52	68.14 ± 11.26	<0.001
Females, n (%)	2979 (37.4)	924 (46.4)	819 (41.1)	689 (34.6)	547 (27.5)	<0.001
High school or above, n (%)	2230 (28.0)	567 (28.5)	560 (28.1)	574 (28.8)	529 (26.6)	0.270
Current smoking, n (%)	2209 (27.7)	492 (24.7)	593 (29.8)	573 (28.8)	551 (27.7)	0.077
Current drinking, n (%)	1666 (20.9)	372 (18.7)	427 (21.4)	422 (21.2)	445 (22.4)	0.008
Lack of exercise, n (%)	5664 (71.1)	1412 (70.9)	1398 (70.2)	1416 (71.0)	1438 (72.3)	0.275
Body mass index, kg/m^2^	24.51 ± 3.52	24.32 ± 3.37	24.62 ± 3.73	24.57 ± 3.54	24.52 ± 3.41	0.119
History of stroke, n (%)	2501 (31.4)	560 (28.1)	589 (29.6)	635 (31.9)	717 (36.0)	<0.001
History of CHD, n (%)	779 (9.8)	166 (8.3)	180 (9.0)	175 (8.8)	258 (13.0)	<0.001
History of AF, n (%)	583 (7.3)	108 (5.4)	114 (5.7)	135 (6.8)	226 (11.4)	<0.001
History of hypertension, n (%)	4757 (59.7)	1102 (55.3)	1181 (59.3)	1243 (62.4)	1231 (61.9)	<0.001
History of diabetes mellitus, n (%)	2007 (25.2)	586 (29.4)	516 (25.9)	465 (23.3)	440 (22.1)	<0.001
Dyslipidemia, n (%)	4498 (56.5)	1163 (58.4)	1110 (55.8)	1092 (54.8)	1133 (56.9)	0.285
Family history of stroke, n (%)	3931 (49.3)	920 (46.2)	984 (49.4)	1019 (51.1)	1008 (50.7)	0.003
Admission mRS (mean ± SD)	1.72 ± 1.53	1.48 ± 1.49	1.61 ± 1.50	1.72 ± 1.51	2.08 ± 1.57	<0.001
Admission NIHSS (median (IQR))	2 (1, 4)	2 (1, 4)	2 (1, 4)	2 (1, 4)	3 (1, 6)	<0.001
IVT, n (%)	683 (8.6)	152 (7.6)	158 (7.9)	177 (8.9)	196 (9.8)	0.007
EVT, n (%)	106 (1.3)	18 (0.9)	24 (1.2)	25 (1.3)	39 (2.0)	0.005
Antihypertensive agents, n (%)	4515 (56.7)	1170 (58.7)	1120 (56.3)	1096 (55.0)	1129 (56.7)	0.143
Hypoglycaemic agents, n (%)	1804 (22.6)	556 (27.9)	457 (23.0)	406 (20.4)	385 (19.3)	<0.0001
Lipid-lowering agents, n (%)	7280 (91.4)	1785 (89.6)	1805 (90.7)	1862 (93.4)	1828 (91.9)	0.001
SBP, mm Hg (mean ± SD)	148.16 ± 18.16	147.57 ± 17.28	147.71 ± 17.59	148.50 ± 18.47	148.86 ± 19.22	0.010
DBP, mm Hg (mean ± SD)	84.55 ± 10.58	83.86 ± 9.85	84.38 ± 10.12	84.85 ± 10.88	85.11 ± 11.36	<0.0001
FBG, mmol/L (median (IQR))	5.70 (5.00, 7.30)	5.80 (5.00, 7.70)	5.70 (5.00, 7.30)	5.70 (5.00, 7.00)	5.70 (5.00, 7.20)	0.018
TG, mmol/L (mean ± SD)	1.76 ± 1.73	1.82 ± 2.16	1.75 ± 1.55	1.78 ± 1.56	1.69 ± 1.58	0.030
TC, mmol/L (mean ± SD)	4.89 ± 2.57	5.08 ± 3.87	4.87 ± 2.14	4.88 ± 2.03	4.72 ± 1.65	<0.001
LDL-C, mmol/L (mean ± SD)	2.96 ± 1.30	3.00 ± 1.15	2.96 ± 1.16	3.00 ± 1.54	2.89 ± 1.32	0.043
HDL-C, mmol/L (mean ± SD)	1.25 ± 1.01	1.30 ± 1.17	1.19 ± 0.54	1.30 ± 1.28	1.21 ± 0.87	0.076
TT, s (median (IQR))	17.00 (15.40, 18.80)	17.00 (15.80, 19.60)	16.90 (15.25, 18.50)	16.70 (15.20, 18.10)	16.70 (15.20, 18.10)	<0.001
PT, s (median (IQR))	12.23 (11.00, 13.20)	12.20 (11.00, 13.30)	12.30 (11.00, 13.20)	12.30 (11.10, 13.30)	12.30 (11.10, 13.30)	0.042
Cr, μmol/L	76.08 ± 44.92	68.21 ± 31.96	71.56 ± 26.25	78.09 ± 55.26	86.48 ± 55.80	<0.001
SUA, μmol/L	303.23 ± 106.13	216.99 ± 97.92	290.39 ± 69.27	327.09 ± 77.59	378.50 ± 104.03	<0.001
Lymphocyte count ×10^9^/L (median (IQR))	1.68 (1.25, 2.12)	2.38 (1.90, 3.00)	1.83 (1.61, 2.17)	1.53 (1.28, 1.80)	1.09 (0.82, 1.30)	<0.001
TOAST classification						
LAA,n (%)	3385 (42.5)	810 (40.7)	803 (40.3)	582 (42.7)	920 (46.2)	0.011
CE,n (%)	328 (4.1)	60 (3.0)	70 (3.5)	55 (2.8)	143 (7.2)	
SAO,n (%)	3530 (44.3)	989 (49.6)	922 (46.3)	889 (44.6)	730 (36.7)	
SOE,n (%)	36 (0.5)	5 (0.3)	8 (0.4)	9 (0.5)	14 (0.7)	
SUE,n (%)	687 (8.6)	128 (6.4)	188 (9.4)	188 (9.4)	183 (9.2)	

AF – atrial fibrillation, CE – cardioembolism, CHD – coronary heart disease, Cr – creatinine, DBP – diastolic blood pressure, EVT – endovascular treatment, FBG – fasting blood glucose, HDL-C – high-density lipoprotein cholesterol, IQR – interquartile range, IVT – intravenous thrombolysis, LAA – large-artery atherosclerosis, LDL-C – low-density lipoprotein cholesterol, NIHSS – National Institutes of Health Stroke Scale, PT – prothrombin time, s – seconds, SAO – small-artery occlusion, SBP – systolic blood pressure, SD – standard deviation, SOE – stroke of other determined aetiology, SUA – serum uric acid, SUE – stroke of undetermined aetiology TC – total cholesterol, TG – triglyceride, TOAST – Trial of Org 10172 in Acute Stroke Treatment, TT – thrombin time, ULR – serum uric acid to lymphocyte count ratio

### The relationship between ULR and different clinical outcomes of stroke

#### Kaplan-Meier curves comparing the incidence of different clinical outcome across the ULR quartile

All 7966 AIS patients included at baseline were enrolled in the survival analysis. The Kaplan-Meier curve results showed that there were significant differences in the incidence rates of adverse stroke outcomes among the groups (all inter-group log-rank *P* < 0.0001), with group Q4 (ULR≥252.5) having a relatively higher incidence rate.

**Figure 2:**
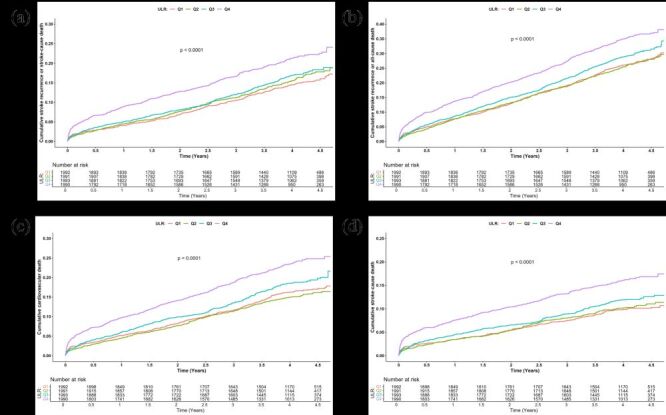
Kaplan-Meier curves of 7966 AIS patients with follow-up data. Comparisons of the cumulative incidence curves between (**Panel A**) stroke recurrence or stroke-cause death, (**Panel B**) stroke recurrence or all-cause death, (**Panel C**) cardiovascular death, (**Panel D**) stroke-cause death in patients with ischemic stroke.

Over time, the survival curve of Group Q4 was observed to rise most rapidly, indicating the highest event rate in this group (**Figure 2**). In comparison, the event rates in Groups Q1, Q2, and Q3 were relatively lower, with their incidence curves rising more gradually. The differences were statistically significant, as indicated by the log-rank test (all *P*-values for comparisons were <0.0001) (**Figure 2**).

#### Cox proportional hazards models exploring the association between the ULR and the risk of different clinical outcomes

During the period of 4.08 years (IQR = 3.35, 4.43) a total of 1311 (16.5%) stroke recurrence or stroke-cause death events, 2370 (29.8%) stroke recurrence or all-cause death events, 1229 (15.4%) CVD death events, and 910 (11.4%) stroke-cause death events occurred. With the increase in ULR quartiles, the incidence rates (per 1000 person-years) of stroke recurrence or stroke-cause death, stroke recurrence or all-cause death, CVD death, and stroke-cause death events significantly increased, ranging from 8.69, 16.54, 9.78, and 5.82 in the Q1 group to 12.24, 21.70, 14.04, and 9.09 in the Q4 group, respectively (**Table 2**).

**Table 2:** Association between quartiles of ULR with the risk of clinical outcomes

Quartiles of ULR	Cases, n	Incidence rate^a^*	Model 1†	*P-*value	Model 2‡	*P-*value	Model 3§	*P-*value
**Stroke recurrence or stroke-cause death**								
Per 1 SD increase			1.12 (1.08, 1.16)	<0.0001	1.08 (1.04, 1.13)	<0.0001	1.07 (1.02, 1.11)	0.002
Q1 (≤127.8)	283	8.69 (8.61, 10.03)	Reference		Reference		Reference	
Q2 (127.9, 179.0)	309	9.49 (8.57, 11.53)	1.10 (0.94, 1.30)	0.235	1.12 (0.95, 1.32)	0.171	1.08 (0.92, 1.27)	0.356
Q3 (179.1, 252.4)	320	9.82 (9.08, 11.99)	1.16 (0.99, 1.36)	0.066	1.11 (0.94, 1.30)	0.222	1.05 (0.90, 1.24)	0.534
Q4(≥252.5)	399	12.24 (11.32, 14.95)	1.55 (1.33, 1.81)	<0.0001	1.37 (1.17, 1.60)	<0.0001	1.21 (1.04, 1.42)	0.016
**Stroke recurrence or all-cause death**								
Per 1 SD increase			1.10 (1.07, 1.13)	<0.0001	1.04 (1.01, 1.07)	0.023	1.02 (1.00, 1.08)	0.208
Q1 (≤127.8)	539	16.54 (15.29, 20.19)	Reference		Reference		Reference	
Q2 (127.9, 179.0)	528	16.21 (14.98, 19.79)	1.00 (0.88, 1.12)	0.862	1.01 (0.90, 1.14)	0.866	0.98 (0.87, 1.11)	0.766
Q3 (179.1, 252.4)	596	18.29 (16.90, 22.36)	1.14 (1.01, 1.28)	0.031	1.07 (0.95, 1.20)	0.279	1.03 (0.92, 1.16)	0.601
Q4(≥252.5)	707	21.70 (20.07, 26.50)	1.45 (1.29, 1.62)	<0.0001	1.22 (1.09, 1.37)	0.001	1.12 (1.00, 1.25)	0.060
**Cardiovascular death**								
Per 1 SD increase			1.12 (1.09, 1.16)	<0.0001	1.05 (1.02, 1.09)	0.006	1.04 (1.00, 1.08)	0.072
Q1 (≤127.8)	318	9.78 (8.99, 11.91)	Reference		Reference		Reference	
Q2 (127.9, 179.0)	294	9.04 (8.31, 11.02)	0.93 (0.79, 1.09)	0.351	0.95 (0.81, 1.12)	0.536	0.91 (0.78, 1.07)	0.255
Q3 (179.1, 252.4)	361	11.11 (10.21, 13.54)	1.16 (1.00, 1.35)	0.051	1.09 (0.94, 1.27)	0.257	1.05 (0.90, 1.22)	0.564
Q4(≥252.5)	456	14.04 (12.89, 17.10)	1.56 (1.35, 1.80)	<0.0001	1.30 (1.13, 1.51)	<0.0001	1.16 (1.00, 1.34)	0.048
**Stroke-cause death**								
Per 1 SD increase			1.14 (1.10, 1.19)	<0.0001	1.09 (1.04, 1.14)	<0.0001	1.07 (1.03, 1.12)	0.002
Q1 (≤127.8)	187	5.82 (5.36, 7.09)	Reference		Reference		Reference	
Q2 (127.9, 179.0)	197	6.06 (5.58, 7.38)	1.06 (0.86, 1.29)	0.599	1.07 (0.88, 1.31)	0.494	1.02 (0.83, 1.24)	0.883
Q3 (179.1, 252.4)	224	6.89 (6.35, 8.40)	1.22 (1.01, 1.48)	0.043	1.15 (0.95, 1.40)	0.155	1.08 (0.89, 1.31)	0.438
Q4 (≥252.5)	302	9.09 (8.56, 11.32)	1.74 (1.45, 2.09)	<0.0001	1.49 (1.23, 1.79)	<0.0001	1.26 (1.05, 1.52)	0.015

BMI – body mass index, Cr – serum creatinine, EVT – endovascular therapy, FBG – fasting blood glucose, IVT – intravenous thrombolysis, mRS – modified rankin scale, SBP – systolic blood pressure, SD – standard deviation, ULR – serum uric acid to lymphocyte count ratio

*Incidence^a^ rate per 1000 person-years.

†Model 1: unadjusted.

‡Model 2: adjusted for age, sex, education, current drinking, current smoking, lack of exercise, BMI, SBP, FBG, dyslipidaemia, and Cr.

§Model 3: further adjusted for prior history of stroke, IVT, EVT, and admission mRS.

We investigated the relationship between admission ULR and various clinical outcomes. As expected, after multivariate adjustment, participants in the Q4 group still had a 21% higher risk of stroke recurrence or stroke-cause death compared to the Q1 group (Q4 *vs*. Q1: HR = 1.21; 95% CI = 1.04, 1.42, *P* = 0.016). Similarly, after multivariate adjustment, the risk of stroke recurrence or stroke-cause death per SD increase in ULR was (Per 1 SD increase *vs*. Q1: HR = 1.07; 95% CI = 1.02, 1.11, *P* = 0.002) still showing a significant positive correlation. The serum uric acid to lymphocyte ratio was also associated with CVD death (Q4 *vs*. Q1: HR = 1.16; 95% CI = 1.00, 1.34, *P* = 0.048), but the association was attenuated to non-significance per SD increase in ULR (Per 1 SD increase *vs*. Q1: HR = 1.04; 95% CI = 1.00, 1.08, *P* = 0.072). In contrast, the association between ULR and stroke-cause death was not weakened either by multivariate adjustment (Q4 *vs*. Q1: HR = 1.26; 95% CI = 1.05, 1.52, *P* = 0.015) or per SD change (Per 1 SD increase *vs*. Q1: HR = 1.26; 95% CI = 1.05, 1.52, *P* = 0.015). Conversely, the association between ULR and stroke recurrence or all-cause death did not reach statistical significance, whether by multivariate adjustment (Q4 *vs*. Q1: HR = 1.12; 95% CI = 1.00, 1.15, *P* = 0.060) or per SD change (Per 1 SD increase *vs*. Q1: HR = 1.02; 95% CI = 1.00, 1.08, *P* = 0.208) (**Table 2****)**.

#### Dose-response association between the ULR and the risk of different clinical outcomes

**Figure 3:**
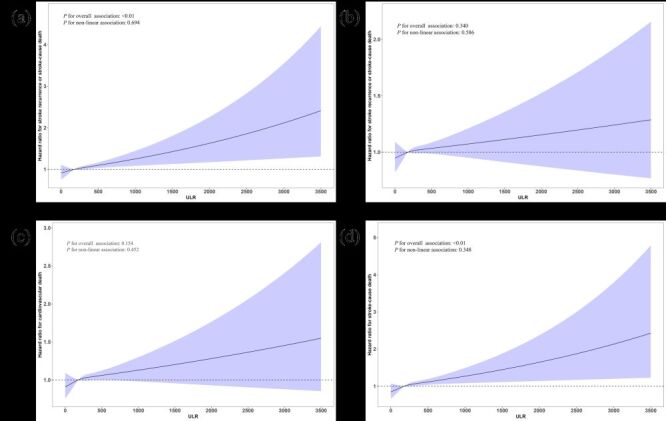
HRs and 95% CIs for ULR with risk of: (**Panel A**) stroke recurrence or stroke-cause death, (**Panel B**) stroke recurrence or all-cause death, (**Panel C**) cardiovascular death, (**Panel D**) stroke-cause death. Adjusted for age, sex, education, current drinking, current smoking, lack of exercise, BMI, SBP, FBG, dyslipidemia, Cr, history of stroke, IVT, EVT, and admission mRS. BMI – body mass index, Cr – serum creatinine, EVT – endovascular therapy, FBG – fasting blood glucose, IVT – intravenous thrombolysis, mRS – modified rankin scale, SBP – systolic blood pressure, ULR – serum uric acid to lymphocyte count ratio.

The results of the multivariate-adjusted spline regression model showed a linear association between ULR and stroke recurrence or stroke-cause death (*P* for overall association <0.01; *P* for nonlinear association = 0.694) (**Figure 3****,** Panel A). Similarly, there was a significant linear association for CVD death (*P* for overall association = 0.154; *P* for nonlinear association = 0.452) (**Figure 3**, Panel C) and stroke-cause death (*P* for overall association <0.01; *P* for nonlinear association = 0.348) (**Figure 3**, Panel D). However, there was no significant association between ULR and stroke recurrence or all-cause mortality (*P* for overall association = 0.340; *P* for nonlinear association = 0.586) (**Figure 3**, Panel B).

### Mediation analysis

Given the significant association between ULR and the risk of stroke recurrence or death, we conducted a mediation analysis to evaluate the potential mechanisms of this association. Potential mediators that might play a role in the relationship between ULR and stroke recurrence or death include BMI, SBP, DBP, FBG, TC, TG, LDL-C, HDL-C, and Cr, with adjustments for age, sex, education, current drinking, and current smoking.

The mediation analysis results revealed that the association between ULR and stroke recurrence or stroke-cause death was primarily mediated through DBP (βindir = 0.0010, mediation proportion (MP) = 8.53%) and SBP (βindir = 0.0004, MP = 3.59%) in a positive correlation manner (**Table 3**). Other factors such as BMI, FBG, TC, TG, LDL-C, HDL-C, and Cr did not show significant mediation effects in the association between ULR and stroke recurrence or stroke-cause death.

**Table 3:** Direct and indirect effects of ULR quartile on stroke recurrence or stroke-cause death and proportion mediated by laboratory indicators*

Potential mediators	Direct effect		Indirect effect		Proportion mediated (%)	*P-*value
	**β_dir_, (95% CI)**	***P-*value**	**β_indir_, (95% CI)**	***P-*value**	
BMI	0.0110 (0.0046, 0.0200)	<0.001	0.0000(−0.0003, 0.0000)	0.6600	−0.32	0.6600
SBP	0.0105 (0.0041, 0.0200)	<0.001	0.0004 (0.0000, 0.0001)	0.0120	3.59	0.0120
DBP	0.0101 (0.0036, 0.0200)	<0.001	0.0010(0.0000, 0.0005)	0.0000	8.53	<0.001
FBG	0.0111 (0.0047, 0.0200)	<0.001	−0.0003 (−0.0007, 0.0000)	0.1800	−2.28	0.1800
TC	0.0109 (0.0044, 0.0200)	<0.001	0.0000 (−0.0002, 0.0000)	0.7400	0.24	0.7400
TG	0.0110 (0.0045, 0.200)	<0.001	−0.0000 (−0.0001, 0.0000)	0.8400	−0.03	0.8400
HDL-C	0.0109 (0.0044, 0.0200)	<0.001	0.0000 (−0.0001, 0.0000)	0.5300	0.24	0.5300
LDL-C	0.0110 (0.0045, 0.0200)	<0.001	−0.0000 (−0.0001, 0.0000)	0.8600	−0.05	0.8600

BMI – body mass index, FBG – fasting blood glucose, HDL-C – high-density lipoprotein cholesterol, LDL-C – low-density lipoprotein cholesterol, SBP – systolic blood pressure, TC – total cholesterol, TG – triglyceride, ULR – serum uric acid to lymphocyte count ratio

*Adjusted for age, sex, education, current drinking, current smoking.

## DISCUSSION

Our study prospectively investigated the relationship between ULR (a novel inflammatory biomarker) at the time of hospital admission in patients with AIS and the risk of stroke recurrence and death post IS, with the aim of reducing the incidence of adverse outcomes in stroke patients. The results demonstrated that after adjusting for confounding risk factors, there was a significant correlation between ULR and the risk of stroke recurrence or stroke-cause death, CVD death, and stroke-cause death. Additionally, the study found that patients with higher quartiles of ULR had a significantly increased risk of recurrence and death, and there was a linear association with various adverse outcomes.

The relative risk of stroke recurrence or stroke-cause death in the Q4 group was 21% higher than that in the Q1 group, and the stable negative association between ULR and clinical outcomes of IS was not affected by age and sex, with its increase being significantly correlated with a rise in stroke risk or severity (Table S1 in the **Online Supplementary Document**). This association is inconsistent with the findings of a previous study by Tian et al. [18], which showed that ULR was significantly associated with the risk of HS in Chinese individuals, and that the application of ULR (a novel biomarker) could enhance the risk stratification capability for the primary prevention of HS. However, our study focused on the prevention of adverse outcomes in patients post IS, and included patients who had already experienced an AIS at baseline, with a higher proportion of female participants and unique population characteristics in Liaoning Province. Therefore, the discrepancy between our findings and those of Tian et al. [18] may be attributed to differences in sample size, distinct characteristics of the study populations, the presence of potential confounding factors, and the use of different statistical methodologies.

Nevertheless, our findings indicate that ULR may serve as a relatively stable risk prediction marker for adverse outcomes following IS and may hold potential for application in secondary prevention of IS. Compared with single inflammatory indicators such as SUA, ULR may provide a more integrated reflection of both inflammatory and immune status. We attempt to explain the underlying mechanisms: First, SUA, as an independent influencing factor, promotes inflammatory responses through various mechanisms such as inducing vascular endothelial dysfunction, promoting inflammatory reactions, and increasing oxidative stress [21–23], thereby increasing the risk of stroke [24–26]. SUA possesses dual roles as both an oxidant and an antioxidant [11]; while the oxidant role increases the risk of adverse stroke outcomes [27], the antioxidant role can exert anti-inflammatory effects by scavenging free radicals, chelating transition metal ions, and maintaining redox balance [28,29]. However, this antioxidant effect is not unconditional [30,31], necessitating further research into the clinical significance of SUA.

Second, the inflammatory cascade triggered by cerebral ischemia, immune regulation imbalance, and increased stress hormones lead to a reduction in lymphocyte count [15]. The role of lymphocytes in cerebral ischemia is dual-faceted: they can promote neuroprotection and repair by secreting anti-inflammatory cells and antibodies through regulatory T cells (Tregs), Th2 cells, and B cells, but they may also exacerbate inflammation, disrupt the blood-brain barrier, and release neurotoxic substances damaging brain tissue through Th1 cells and cytotoxic T cells, necessitating cautious evaluation of lymphocytes as a prognostic marker for IS [15,32].

An elevated ULR reflects an increase in SUA or a decrease in lymphocyte count, both of which are associated with high levels of inflammation. SUA damages lymphocytes through pro-inflammatory and oxidative stress pathways, weakening immune regulation and leading to persistent inflammation exacerbation [33,34]. Therefore, ULR, as a comprehensive indicator, is more suitable for objectively assessing the risk of recurrence and death after IS.

In addition, mediation analysis showed DBP and SBP mediated the link between ULR and stroke recurrence or stroke-cause death. Elevated SBP raises arterial shear stress, promotes atherosclerosis, causes vascular stenosis and plaque instability, increasing thrombosis and stroke risk [35]. Hypertension may also induce cerebral small vessel disease, raising haemorrhage and infarction risk [36,37]. Conversely, very low DBP can cause insufficient cerebral perfusion, especially with atherosclerosis, leading to ischemia and reflecting vascular stiffness and endothelial dysfunction [38]. These factors may also increase the risk of stroke and its adverse outcomes.

Our study has key strengths. First, it is a large, prospective study examining ULR’s link to IS recurrence and death. Second, year-round enrollment reduced seasonal bias. Third, we added IVT, EVT, and mRS data to support ULR’s risk stratification role. Fourth, ULR combines SUA and lymphocyte count, reflecting inflammation and immunity more fully. ULR is simple, accessible, and cost-effective for early risk assessment and personalised stroke care, especially in resource-limited settings. With high stroke burden and limited advanced testing in LMICs, ULR based on routine blood tests holds strong clinical promise. Further research should validate its use and cost-effectiveness in diverse health care settings.

However, several limitations exist. First, the 4.08-year median follow-up may not capture all long-term or rare outcomes. Second, stroke recurrence could be underreported due to loss to follow-up, recall bias, and incomplete records, potentially underestimating recurrence rates and affecting ULR associations. Third, the study’s focus on Liaoning Province, China, limits generalisability due to unique local factors, requiring validation in other populations. Fourth, ULR, combining SUA and lymphocyte count, better reflects inflammatory and immune balance than single markers, enhancing prognostic value.

## CONCLUSIONS

ULR, as a novel inflammatory marker, has a stable negative association with the clinical outcomes of IS. ULR has the potential to become an effective tool for secondary prevention of IS risk in clinical practice, but its application value needs to be further confirmed by research. Future studies should focus on exploring the biological mechanisms of ULR in depth and validating its predictive capabilities in different populations, so as to better realise the individualised prediction and treatment of stroke.

## Additional material


Online Supplementary Document
