# Supplementary material for: Relationship between admission serum uric acid to lymphocyte ratio and the risk of ischemic stroke recurrence and death: a prospective study
Source: J Glob Health. 2025 Sep 12;15:04240. doi: 10.7189/jogh.15.04240 (PMC12426691; doi:10.7189/jogh.15.04240)
Supplement: Online Supplementary Document [file jogh-15-04240-s001.pdf]

Supplement to: Song C, Li G, Tian Y, Jing L, Chen D, Wang W, Shi Y, Wang D, Li D, Hao W, Xing L, Liu S. Relationship between admission serum uric acid to lymphocyte ratio and the risk of ischemic stroke recurrence and death: a prospective study. J Glob Health. 2025;15:04240.

Supplementary Table 1. Sex-stratified Hazard Ratios of Outcomes by ULR Quartiles

Subgroups	HR (95%)	P Value	P for interaction
Stroke recurrence or stroke-cause death: Female			0.191
Q1 (≤127.8)	Reference		
Q2 (127.9 to 179.0)	1.22(0.94,1.58)	0.128	
Q3 (179.1 to 252.4)	1.37(1.06,1.77)	0.017	
Q4(≥252.5)	1.34(1.02,1.75)	0.035	
Stroke recurrence or stroke-cause death: Male			
Q1 (≤127.8)	Reference		
Q2 (127.9 to 179.0)	0.99(0.80,1.22)	0.921	
Q3 (179.1 to 252.4)	0.89(0.72,1.09)	0.247	
Q4(≥252.5)	1.12(0.92,1.35)	0.266	
Stroke recurrence or all-cause death: Female			0.283
Q1 (≤127.8)	Reference		
Q2 (127.9 to 179.0)	1.00(0.83,1.21)	0.997	
Q3 (179.1 to 252.4)	1.19(0.99,1.43)	0.068	
Q4(≥252.5)	1.11(0.92,1.35)	0.288	
Stroke recurrence or all-cause death: Male			
Q1 (≤127.8)	Reference		
Q2 (127.9 to 179.0)	0.97(0.83,1.13)	0.671	
Q3 (179.1 to 252.4)	0.94(0.80,1.09)	0.398	
Q4(≥252.5)	1.09(0.94,1.26)	0.251	
Cardiovascular death: Female			0.571
Q1 (≤127.8)	Reference		
Q2 (127.9 to 179.0)	0.90(0.71,1.15)	0.401	
Q3 (179.1 to 252.4)	1.15(0.91,1.45)	0.233	
Q4(≥252.5)	1.04(0.82,1.33)	0.758	
Cardiovascular death: Male			
Q1 (≤127.8)	Reference		
Q2 (127.9 to 179.0)	0.93(0.75,1.15)	0.491	
Q3 (179.1 to 252.4)	0.98(0.80,1.21)	0.864	
Q4(≥252.5)	1.21(1.00,1.46)	0.048	
Stroke death: Female			
Q1 (≤127.8)	Reference		0.930
Q2 (127.9 to 179.0)	1.09(0.80,1.48)	0.596	
Q3 (179.1 to 252.4)	1.30(0.96,1.76)	0.088	
Q4(≥252.5)	1.22(0.89,1.67)	0.224	
Stroke death: Male			
Q1 (≤127.8)	Reference		
Q2 (127.9 to 179.0)	0.97(0.74,1.26)	0.800	
Q3 (179.1 to 252.4)	0.96(0.74,1.24)	0.730	

Q4(≥252.5)	1.25(0.98,1.58)	0.068

Adjusted for age, sex, education, current drinking, current smoking, lack of exercise, BMI, SBP, FBG, dyslipidemia, Cr, history of stroke, IVT, EVT, and admission mRS.
